# Family A DNA Polymerase Phylogeny Uncovers Diversity and Replication Gene Organization in the Virioplankton

**DOI:** 10.3389/fmicb.2018.03053

**Published:** 2018-12-14

**Authors:** Daniel J. Nasko, Jessica Chopyk, Eric G. Sakowski, Barbra D. Ferrell, Shawn W. Polson, K. Eric Wommack

**Affiliations:** ^1^Delaware Biotechnology Institute, University of Delaware, Newark, DE, United States; ^2^School of Public Health, University of Maryland, College Park, MD, United States; ^3^Department of Environmental Health and Engineering, Johns Hopkins University, Baltimore, MD, United States

**Keywords:** metagenomics, viral ecology, bacteriophage, genome to phenome, bioinformatics

## Abstract

Shotgun metagenomics, which allows for broad sampling of viral diversity, has uncovered genes that are widely distributed among virioplankton populations and show linkages to important biological features of unknown viruses. Over 25% of known dsDNA phage carry the DNA polymerase I (*polA*) gene, making it one of the most widely distributed phage genes. Because of its pivotal role in DNA replication, this enzyme is linked to phage lifecycle characteristics. Previous research has suggested that a single amino acid substitution might be predictive of viral lifestyle. In this study Chesapeake Bay virioplankton were sampled by shotgun metagenomic sequencing (using long and short read technologies). More *polA* sequences were predicted from this single viral metagenome (virome) than from 86 globally distributed virome libraries (ca. 2,100, and 1,200, respectively). The PolA peptides predicted from the Chesapeake Bay virome clustered with 69% of PolA peptides from global viromes; thus, remarkably the Chesapeake Bay virome captured the majority of known PolA peptide diversity in viruses. This deeply sequenced virome also expanded the diversity of PolA sequences, increasing the number of PolA clusters by 44%. Contigs containing *polA* sequences were also used to examine relationships between phylogenetic clades of PolA and other genes within unknown viral populations. Phylogenic analysis revealed five distinct groups of phages distinguished by the amino acids at their 762 (*Escherichia coli* IAI39 numbering) positions and replication genes. DNA polymerase I sequences from Tyr762 and Phe762 groups were most often neighbored by ring-shaped superfamily IV helicases and ribonucleotide reductases (RNRs). The Leu762 groups had non-ring shaped helicases from superfamily II and were further distinguished by an additional helicase gene from superfamily I and the lack of any identifiable RNR genes. Moreover, we found that the inclusion of ribonucleotide reductase associated with PolA helped to further differentiate phage diversity, chiefly within lytic podovirus populations. Altogether, these data show that DNA Polymerase I is a useful marker for observing the diversity and composition of the virioplankton and may be a driving factor in the divergence of phage replication components.

## Introduction

Bacteriophage are the most abundant biological entities on the planet, driving food web-dynamics, nutrient cycling, and host composition. The phage present in the Chesapeake Bay alone would dwarf the number of sand grains in all the world’s beaches and deserts, an estimated 7.5 × 10^18^ grains versus 1.7 × 10^24^ viral particles (Wommack et al., 1992). Although estimates of global viral diversity vary widely (Cesar Ignacio-Espinoza et al., 2013) it is a certainty that within the profuse abundance of viral particles lies an expansive genetic diversity (Bench et al., 2007; Paez-Espino et al., 2016). However, the task of understanding the population-scale structure of the virioplankton is complicated by the lack of a universally conserved marker gene for cataloging viral diversity, akin to the 16S rRNA gene in bacteria (Woese and Fox, 1977). In the face of this challenge, several structural and functional genes have been used as markers of phage diversity for specific phage groups, including structural genes g20 and g23 for myoviruses and DNA Polymerase A for podoviruses (Breitbart et al., 2004b; Jameson et al., 2011; Chow and Fuhrman, 2012).

Family A DNA polymerase is the primary enzyme responsible for phage genome replication (Doublie et al., 1998) within the ca. 25% of all known double-stranded DNA (dsDNA) phage known to carry this gene (Wommack et al., 2015). Using PCR based approaches, *polA* genes in T7-like podoviruses have been identified across multiple environments and in some cases exhibit environmental specificity (Labonté et al., 2009). Prior *in vitro* site-directed mutagenesis studies of PolA peptides have found that amino acid substitutions from the wild type phenylalanine at position Phe762 (*Escherichia coli* IAI39 numbering) introduces biochemical changes within the polymerase (Tabor and Richardson, 1987, 1995; Suzuki et al., 2000). A Phe762 to tyrosine substitution (Tyr762) of the coliphage T7 DNA polymerase was shown to produce a more efficient polymerase with substantial increases in the incorporation rate of ddNTPs for enzymes lacking the 3′–5′ exonuclease (Tabor and Richardson, 1995; Astatke et al., 1998). In nature, the selective pressure for the Tyr762 substitution within phage PolA genes is likely higher dNTP incorporation efficiency (Schmidt et al., 2014). Conversely, the Phe762 to leucine mutation (Leu762) in *Thermus aquaticus* Pol A produced a slower but more accurate polymerase (Suzuki et al., 2000). All three PolA 762 types can be commonly observed within the virioplankton and within known tailed phages (Schmidt et al., 2014). A metanalysis of phage genomes indicated that these single amino acid changes in the motif B region of PolA appeared to be linked with phage lifecycle (Schmidt et al., 2014). The higher efficiency of the wt^+^ (Phe762) and Tyr762 mutation occurred only in virulent phages; whereas, the lower efficiency, higher-fidelity Leu762 mutation occurred primarily in temperate phages. Earlier, PCR-based studies of phage populations failed to detect the prevalence of the Leu762 group within the virioplankton, which was likely due to the limitations in the breadth of sequences available in the initial datasets used to design the PCR primers (Breitbart et al., 2004b; Labonté et al., 2009). This illustrates the importance of shotgun metagenomic approaches for capturing the true extent of viral genetic diversity.

Shotgun viral metagenomics makes it possible to observe and analyze random parts of viral genomes across viral populations within an environmental sample without cultivation. This method has been employed to characterize the genetic diversity of environmental phage from a wide variety of environments including: oceans, estuaries, soils, hydrothermal vents, hot springs, and organismal substrates (Breitbart et al., 2004a; Fierer et al., 2007; Schoenfeld et al., 2008; Anderson et al., 2014). Despite the fact that over 60% of predicted open reading frames (ORFs) within a typical virome demonstrate no homology to sequences of known reference genomes, shotgun metagenomics nevertheless continues to be the best means of exploring the composition and diversity of viral communities (Breitbart et al., 2002; Paez-Espino et al., 2016).

As next generation sequencing has advanced in terms of both throughput and read length, so too have assembly algorithms. Longer reads/contigs have improved homology searches (Wommack et al., 2008) and enabled the reconstruction of complete or nearly complete phage genomes (i.e., informative contigs ≥30 Kbp) from metagenome sequence libraries (Iverson et al., 2012; Mizuno et al., 2013; Smits et al., 2014). Among the significant scientific breakthroughs enabled by metagenomics has been the ability to explore genetic linkages that may define the ecological and biological features of unknown viral populations. For instance, within phage genomes *polA* tends to be located in close association with other replication-encoding genes (e.g., helicases, primases, single stranded binding proteins, endo/exonucleases). Given the importance of genome replication to the fitness of a viral population it is possible that “replication modules” can demonstrate strong links to predicting the biological and ecological features of unknown viruses.

This study demonstrates the utility long contigs can provide for investigating viral diversity by using a combined phylogenetic and multi-gene approach. The replisome of unknown bacteriophage populations carrying a *polA* gene was assessed within the framework of an established DNA polymerase I phylogeny. The overarching objective was to build a framework for defining unknown viral populations through the genetic composition of *polA*-containing replisomes. The *polA*-containing contigs revealed the presence of multiple phage populations that were divergent in replisome composition, differences that likely linked with key phenomic characteristics such as phage lifecycle and host preference.

## Materials and Methods

### Metagenomic Libraries

The iron chloride precipitation procedure (John et al., 2011) was used to concentrate viral particles from 50 L of surface water taken from the Smithsonian Environmental Research Center (SERC) within the Chesapeake Bay watershed in December 2012. Briefly, 4.83 g of FeCl_3_ were dissolved in 100 mL of 0.02 μm-filtered water. One milliliter of the FeCl_3_ solution was then added for each 10 L of the 0.2 μm-filtered Chesapeake Bay watershed sample. After a 1 h room temperature incubation, viral particles trapped within FeCl_3_ flocculate were filtered onto 0.8 μm polycarbonate filters (Whatman). FeCl_3_ flocculate trapped on the filter was then resuspended in oxalic acid buffer as previously described (John et al., 2011). DNA was extracted from the viral concentrate using the phenol crack approach as previously described (Marine et al., 2017). Virioplankton DNA was sequenced with one lane of Illumina Hi-Seq (Rapid protocol; PE 2x150 reads) and nineteen single molecule real-time (SMRT) sequencing cells on the PacBio RSII instrument, using both the standard and terminal deoxynucleotidyl transferase (TdT) protocols (Tsai et al., 2016). The Illumina sequence reads are available on NCBI’s Sequence Read Archive (SRA) under experiment SRX2188694. The PacBio reads are also available on the SRA under experiment IDs SRX2194795 and SRX2194790. Additional publicly available virome libraries were obtained from the Metagenomes Online (MgOl)^1^ peptide database (Supplementary File S1).

### Assembly of SERC Metagenome Sequences

Three assembly approaches were used on the SERC virome read libraries (Supplementary Table S1). One assembly was generated with Illumina reads using Celera (version 8.1) (Myers et al., 2000). A second assembly was generated with PacBio long reads using the Hierarchical Genome Assembly Process (HGAP version 3) (Chin et al., 2013). Additionally, a hybrid assembly approach was employed to exploit the accuracy of Illumina and the long-read length of PacBio. PacBio reads ≥1,000 BP were error corrected with unitigs from the Illumina-only Celera assembly using the ECTools pipeline (Lee et al., 2014). The error corrected PacBio reads were then combined with the Illumina-only unitigs and a hybrid assembly of the two datasets was performed using Celera assembler (Myers et al., 2000). The 86 additional virome libraries that were retrieved from MgOl database (see text footnote 1) were previously assembled and thus were ready for immediate analysis.

### Viral DNA Polymerase I Prediction

Open reading frames were predicted for the SERC assembly and each of the MgOl libraries using MetaGeneMark (Zhu et al., 2010). Predicted ORFs were queried against a database of PolA UniRef90 (Suzek et al., 2007) clusters using protein-protein BLAST (BLASTp) (Altschul et al., 1997) using *E*-value ≤1e-5 as a cut-off. Positive hits were filtered based on length (≥200 aa) and then confirmed to be PolA via NCBI’s Conserved Domain BLAST online tool (Marchler-Bauer et al., 2011).

### Alignments and Phylogenetic Trees

A tree comprising both SERC and MgOl PolA peptides was constructed to determine the global diversity of PolA. As the MgOl peptide ORFs were typically much shorter than the SERC peptide ORFs, it was necessary to reduce the PolA region of interest for the analysis to retain as many PolA sequences as possible. Thus, a shorter region (125 aa), corresponding to N675-L799 in the *E. coli* PolA gene product, was extracted from predicted PolA peptides within the SERC and MgOl virioplankton libraries. These sequences were then aligned in MAFFT using the FFT-NS-i × 1000 algorithm (Katoh et al., 2002) and clustered at 75% using the furthest neighbor algorithm in Mothur (Schloss et al., 2009). Cluster representatives were used to build an unrooted maximum likelihood tree with 10 bootstrap replicates using Geneious 6.0.5 (Kearse et al., 2012) with PhyML (Guindon and Gascuel, 2003). Tree branches were colored by cluster member source(s) using Iroki (Moore et al., 2018) and a ring indicating 762-type was created around the tree using Autodesk^®^ Graphic.

Additionally, PolA sequences from the hybrid SERC assembly that were on contigs with neighboring genes were aligned using MAFFT applying the FFT-NS-i × 1000 algorithm. Alignment of each virioplankton PolA with the PolA protein from *E. coli* IAI39 enabled determination of the amino acid residing at the 762 position. Full length PolA domains (*E. coli* I547-Q926) were extracted from the large alignment and clustered at 75% using the furthest neighbor algorithm in Mothur (Supplementary Table S1). An unrooted maximum likelihood tree with 100 bootstrap replicates was generated as previously described. Tree branches were colored by the 762-type and the gene neighbor combinations were added to the figure.

Associations between PolA and several genes involved in DNA replication were assessed using a manual gene neighbor analysis. Among these neighboring genes were ribonucleotide reductases (RNRs), the only enzymes known to be capable of reducing ribonucleotides to deoxyribonucleotides (Lundin et al., 2010). PolA sequences and their neighboring RNR sequences were extracted from contigs. PolA and RNR trees were constructed by aligning each set of sequences with MAFFT using the FFT-NS-i × 1000 algorithm and building the unrooted maximum likelihood trees with 10 bootstrap replicates. These trees were colored according to the PolA 762-type and RNR biochemical class. Nodes were added to the tip of each branch and colored by the neighboring gene’s classification.

To achieve better phylogenetic resolution of these phage populations a concatenated tree of PolA and RNR was also constructed. Both PolA and RNR peptides were extracted from PolA-containing contigs in the SERC PacBio-Illumina hybrid assembly. A 189-aa region, corresponding to N437-S625 in the *E. coli* RNR *nrdA* gene product (Sakowski et al., 2014), was extracted and concatenated with corresponding PolA peptides trimmed to a region of similar length (*E. coli* A784-Q926). Concatenated sequences were aligned with MAFFT using the FFT-NS-i × 1000 algorithm and an unrooted maximum likelihood tree with 10 bootstrap replicates was created as previously described. Branches were colored by RNR classification using Iroki and nodes were added to the tip of each branch and colored by the neighboring PolA 762-type; this was done using Autodesk^®^ Graphic.

### SERC Contig Gene Neighbor Analysis

Predicted ORFs from SERC contigs carrying verified PolA sequences were annotated by homology search using NCBI’s Conserved Domain Database tool (Marchler-Bauer et al., 2011). These contigs were then categorized by the presence of the following genes involved in DNA replication: helicase (UvrD-like, DnaB-like, Gp4-like, RecB-like, SNF2-like) and ribonucleotide reductase (Class I Other, Class II Other, and Class II RTPR), as previously defined (Sakowski et al., 2014; Supplementary Table S2). The Class I Cyano SP ribonucleotide reductase has been recently defined (Harrison et al., 2018) based on Class II Cyano sequences from a previous study (Sakowski et al., 2014; Supplementary Table S2). Contigs from the PacBio-Illumina hybrid assembly with full-length PolA sequences were annotated and sorted into groups based on PolA phylogeny and replication organization. All other genes were sorted based on prevalence within contigs (Supplementary File S2). To estimate the abundance of each gene combination within the SERC metagenome a recruitment of all Illumina reads to the contigs with PolA sequences was performed using Bowtie2 (very sensitive, end-to-end) (Langmead and Salzberg, 2012).

An additional gene neighbor analysis was performed on contigs containing both PolA and RNR sequences. All predicted peptides from all contigs containing a PolA and RNR were clustered using a nearest neighbor approach. This was achieved by performing an all-vs-all BLASTp search of the peptides (*E*-value ≤1e-20) and parsing through results with a script that clustered peptides together based on their hits with other peptides^2^. Clusters that were found in at least half of the contigs of a given clade were identified as “core” clusters for that clade. These clusters were annotated for gene function by searching them against viral Refseq (O’Leary et al., 2016) with BLASTp (*E*-value ≤1e-5). Lastly, each of the core gene clusters for a given clade were labeled according to the known virus demonstrating the best BLAST homology to the PolA and RNR sequences within the clade (i.e., BLAST against viral Refseq with BLASTp; *E*-value ≤1e-5).

## Results

### Deep Sequencing and Hybrid Assembly Provides Largest Yield of Viral PolA Sequences

The hybrid assembly of Illumina and PacBio reads from the SERC sample produced nearly twice as many predicted PolA peptides ≥200 amino acids with a conserved 762 position than either technology alone. In total, 2,095 PolA peptides were produced by the hybrid assembly compared to 1,074 with Illumina-only and 138 with PacBio-only (Supplementary Table S1). Among the hybrid assembly PolA sequences (≥200 aa) there were: 934 Leu762 sequences, 691 Tyr762 sequences, and 420 Phe762 sequences (Table 1). PolA peptides were also predicted from the viral MgOl libraries and binned via the same process as the SERC datasets (Table 1). In total, 1,215 PolA sequences ≥200 amino acids with a conserved 762 position were identified from 86 virome libraries (Table 1). The Leu762 mutation was the most prevalent (624), followed by the Tyr762 (347) and Phe762 (244) mutation types (Table 1).

**Table 1:** Predicted viral PolA sequences (≥200 aa) from hybrid SERC assembly and MgOl libraries.

						DNA polymerase I counts		
**Collection**	**Sphere**	**Libraries**	**Peptides (millions)**	**Phe762**	**Tyr762**	**Leu762**	**Total**	**Normalized (Pol I/1 M peptides)**
MgOl	Aquatic	79	366	236	342	617	1195	4
	Organismal	5	20	6	5	7	18	1
	Terrestrial	2	2	2	0	0	2	0
	Subtotal	86	388	244	347	624	1215	3
SERC	Aquatic	1	186	420	691	934	2045	11


### Global Distribution of PolA Diversity

**FIGURE 1:**
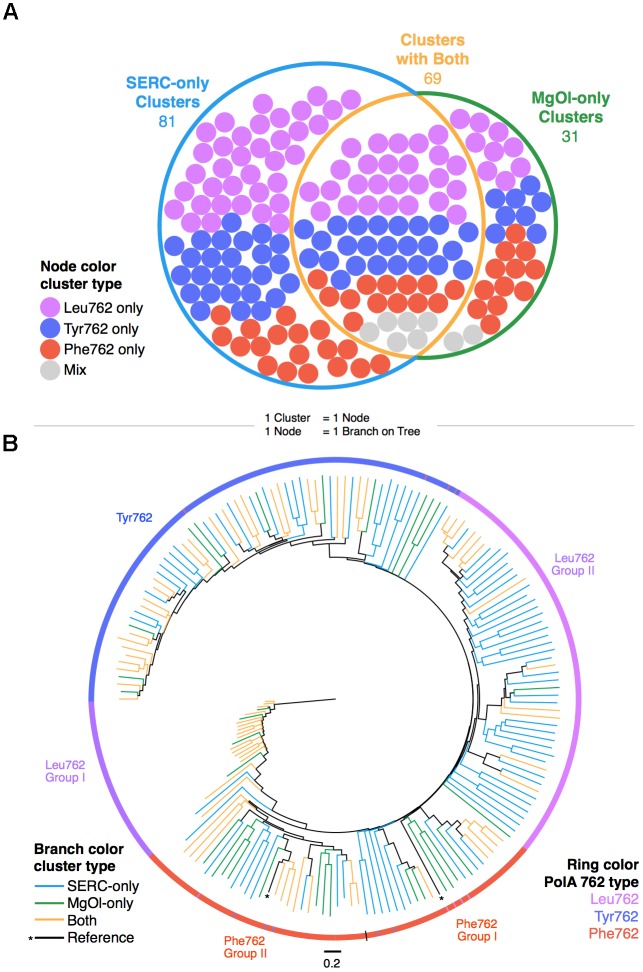
Greater than two-thirds of global PolA diversity captured in a single Chesapeake Bay sample. **(A)** PolA peptide clusters (75% amino acid identity) among the SERC hybrid assembly and MgOl libraries. Each node within the diagram represents one PolA cluster and is colored by 762 position type (purple – Leu762; blue – Tyr762; red – Phe762; gray – mixture of 762 types in a cluster). **(B)** Unrooted maximum likelihood tree with ten bootstrap replicates of partial-length (*E. coli* N675-L799) PolA peptide representative sequences from the SERC hybrid assembly and 86 MgOl libraries clustered at 75%. Line colors represent cluster members source(s), and ring color denotes 762 position grouping based on 762 position residue within the PolA sequences.

The MgOl and SERC PolA sequences formed 181 clusters at 75% amino acid identity (AAI). In total, 100 PolA clusters contained MgOl sequences and 150 PolA clusters contained SERC sequences (Figure 1A). Sixty-nine clusters contained PolA sequences from both the MgOl and SERC libraries; 31 clusters contained only MgOl sequences and, 81 clusters contained only SERC sequences. Each of the 181 PolA sequences representing the 75% AAI clusters (Figure 1A) was placed on an unrooted maximum likelihood (UML) tree (Figure 1B). This tree revealed five groups defined by the 762 position namely: Tyr762; Phe762 Group I and II; and Leu762 Group I and II (Figure 1B).

The Tyr762 group was comprised of 23 SERC-only clusters, 9 MgOl-only clusters, and 26 clusters contained sequences from both the MgOl and SERC datasets. In total, PolA sequences from 14 geographic locations were represented in the Tyr762 group: Chesapeake Bay, Aloha Station (Hawaii), North Sea, Caroline Island, Dry Tortugas (Florida), Gulf of Maine, Mediterranean Sea, Pacific Ocean, Raunefjorden (Norway), Scripps Pier (California), Strait of Georgia, Tampa Bay, Wreck Reef (Puerto Rico), and Octopus Hot Spring (Wyoming).

Phe762 Group I and Phe762 Group II diverged as two distinct superclades that included nine deviant sequences (i.e., the residue at the 762 position was not a Phe in these sequences; indicated by colors deviating from the expected assignment Figure 1B). The Phe762 Group I contained representative sequences from eight SERC-only clusters, four MgOl-only clusters and five clusters containing both SERC and MgOl sequences. Altogether, seven geographic locations were represented in Phe762 Group I: Chesapeake Bay, Dry Tortugas (Florida), East Pacific Rise, Guaymas Basin (Mexico), Gulf of Maine, Pacific Ocean, Caroline Island.

The Phe762 Group II contained representative sequences from eight SERC-only clusters, seven MgOl-only clusters and nine both SERC and MgOl sequences. Ten geographic locations were represented in Phe762 Group II: Chesapeake Bay, Aloha Station (Hawaii), Bear Paw Hot Spring (Wyoming), Dry Tortugas (Florida), Gulf of Maine, Pacific Ocean, Santa Monica Basin, Scripps Pier (California), Octopus Hot Spring (Wyoming), Sargasso Sea.

Leu762 Group I contained representative sequences from only one SERC-only clusters, four MgOl-only clusters and 16 both SERC and MgOl sequences. Thirteen geographic locations were represented in Leu762 Group I: Chesapeake Bay, Aloha Station (Hawaii), North Sea, Dry Tortugas (Florida), East Pacific Rise, Eel River (California), Gulf of Maine, Pacific Ocean, Point Loma (California), Scripps Pier (California), Tampa Bay, Sargasso Sea, and Caroline Island.

Leu762 Group II contained representative sequences from 36 SERC-only clusters, the largest number of SERC-only clusters in any PolA group, one MgOl-only cluster, and 12 both SERC and MgOl sequences. Leu762 Group II was represented in ten different geographic locations: Chesapeake Bay, North Sea, Dry Tortugas (Florida), East Pacific Rise, Gulf of Maine, Pacific Ocean, Reinefjorden (Norway), Scripps Pier (California), Tampa Bay, and Caroline Island.

Overall, there were some geographic locations that had representation from each group. These included: Chesapeake Bay, Dry Tortugas (Florida), Gulf of Maine and Pacific Ocean. Additionally, there were some locations that had representation from only one group: Wreck Reef (Tyr762 group), Strait of Georgia (Tyr762 group), Mediterranean Sea (Tyr762 group), Point Loma (Leu762 Group I), Eel River (Leu762 Group I), Guaymas Basin (Phe762 Group I), Santa Monica Basin (Phe762 Group II), and Bear Paw Hot Spring (Phe762 Group II).

### Gene Neighbor Analysis and Phylogeny Reveal Distinct Replication Organization

**FIGURE 2:**
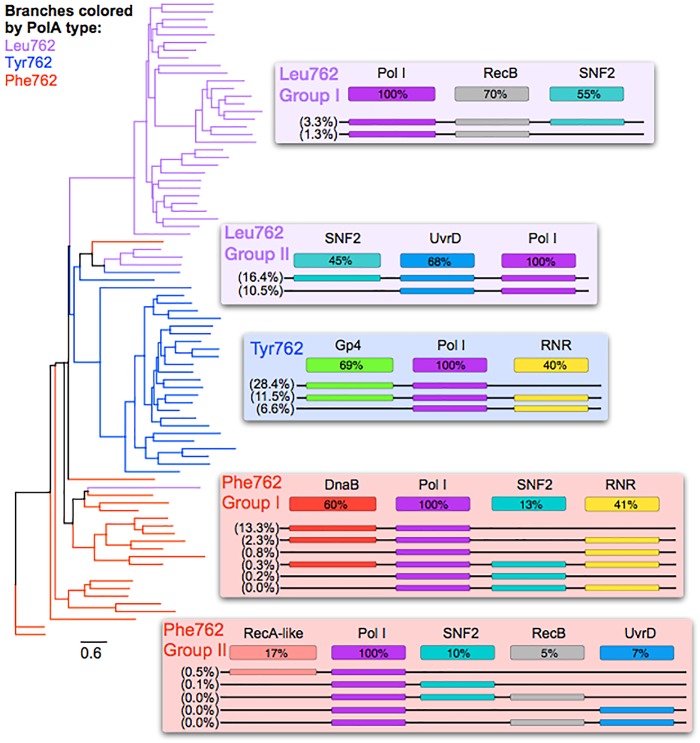
Gene neighbor analysis and phylogeny reveal distinct replication organization. Unrooted maximum likelihood tree with 100 bootstrap replicates of representative sequences from full-length (*E. coli polA* gene I547-Q926) PolA peptide sequences clustered at 75% with predicted replication components. Lines are colored by 762 position residues and grouped by corresponding clade (purple – Leu762; blue – Tyr 762; red – Phe762). The contigs containing the PolA peptide sequences within each group were annotated for adjacent replication components (e.g., helicases RecB-like, Uvrd-like, SNF2-like, and RNR), and representative replisome arrangements and abundances appear to the right of the tree. Replisome labels include the percentage of contigs containing each gene in that clade (written in each gene’s box), and the percentage of read coverage recruited to contigs containing that arrangement (written to the left of each line).

Using only PolA sequences assembled from the hybrid SERC dataset (Supplementary Table S1) a UML tree of 75% AAI representative PolA sequences revealed that the five groups defined in the large tree (Figure 1A) also existed in the SERC dataset (Figure 2). Each group corresponded to different replisome content (i.e., the collection of replication-related genes) and organization, indicating that PolA phylogeny is somewhat predictive of the surrounding replisome gene content. The Tyr762 Group contained 289 PolA sequences forming 27 clusters. A Gp4-like helicase was annotated in 69% and a ribonucleotide-reductase (RNR) in 40% of PolA-containing contigs. Gp4-like helicases and RNR neighbored Tyr762 PolA sequences 34% of the time.

Phe762 Group I contained 124 PolA sequences forming 11 clusters. DnaB-like helicases were downstream of Phe762 Group I PolA sequences 60% of the time. SNF2-like helicases were found with these PolA sequences 13% of the time and either class I or class II RNRs were found with these PolA sequences 41% of the time. Phe762 Group II contained 42 PolA sequences forming eight clusters. Several helicases were found neighboring Phe762 Group II PolA sequences at varying frequencies: RecA-like helicases 17%, SNF2-like helicases 10%, RecB-like helicases 5%, and UvrD-like helicases 7%.

Leu762 Group I contained 119 PolA sequences that formed 31 clusters. Of these PolA sequences 70% had a RecB-like helicase neighboring the PolA gene, while 55% had a neighboring SNF2-like helicase. Both RecB-like and SNF2-like helicases neighbored the PolA sequences 54% of the time. Leu762 Group II contained 247 PolA sequences in three clusters. Among these PolA sequences 45% had a neighboring SNF2-like helicase and 68% have a neighboring UvrD-like helicase. UvrD-like helicases neighboring a Leu762 Group II PolA were always found with an SNF2-like helicase.

Using read recruitment information the abundance of each PolA and replication gene(s) combination was measured. The most abundant combination of PolA and replication gene in the SERC sample was the Tyr762 PolA and Gp4-like helicase (28.4% of reads recruiting to PolA contigs recruit to contigs with this combination). The second and third most abundant combinations were Leu762 Group II PolAs with an SNF2-like helicase and UvrD-like helicase (16.4%) and Phe762 Group I PolAs with a DnaB-like helicase (13.3%).

### Neighboring PolA and RNR Sequences Reveal a More Parsimonious Phylogeny

Within the Illumina-PacBio hybrid assembly, 140 contigs contained full length PolA and RNR sequences. The majority of the PolA-RNR containing contigs encoded Tyr762 PolA sequences (70%), while the remaining PolA sequences were typically Phe762 (28%) with few Leu762 PolA sequences (2%). The majority of RNRs were O_2_-independent class II enzymes (62%) and were further sub-classified as follows according to clades defined in (Sakowski et al., 2014): class II RTPR (46%), class II “Other” (18%). The remaining RNRs were O_2_-dependent class I enzymes in the “Other” clade (28%) and the recently defined Cyano SP clade (10%) (Harrison et al., 2018; Supplementary Table S2). Recent work has shown that the Cyano II clade defined by Sakowski et al. (2014) is not a class II RNR, but is instead a class I RNR (Harrison et al., 2018). As a consequence, the clade was renamed from Cyano II to Cyano SP.

**FIGURE 3:**
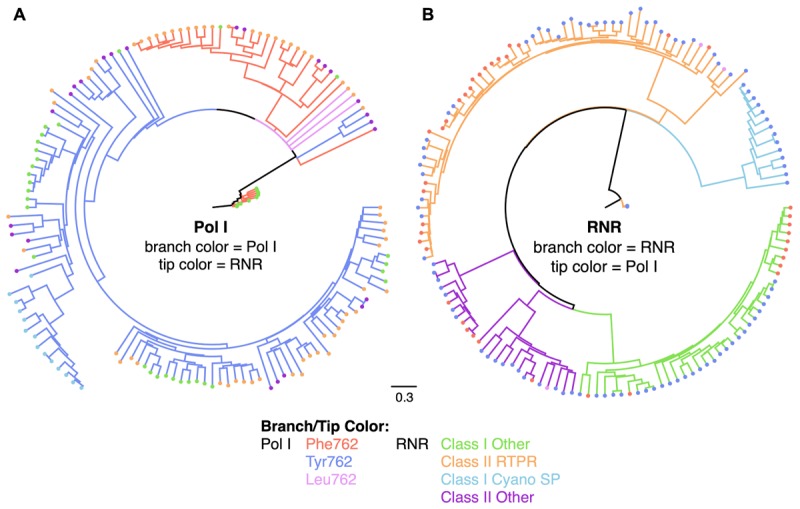
PolA and RNR sequences offer distinct evolutionary histories of lytic virioplankton. Unrooted maximum likelihood trees with 10 bootstrap replicates of **(A)** PolA (*E. coli polA* gene A784-Q926) and **(B)** RNR (*E. coli nrdA* gene N437-S625) sequences. Branches in phylogram **A** are colored by PolA type (purple – Leu762; blue – Tyr 762; red – Phe762) and in phylogram **B** by RNR types (green – Class I Other; orange – Class II RTPR; light blue – Class I Cyano SP; purple – Class II Other). Nodes at the tips of the PolA tree **(A)** are colored by the neighboring RNR types while nodes at the tips of the RNR tree **(B)** are colored by the neighboring PolA types. Color code for the nodes are the same as that of the branches.

Phylogenetic analysis independently examining PolA and RNR peptides revealed two different evolutionary histories (Figure 3). PolA amino acid sequences (annotated by PolA 762 type and the neighboring RNR class) clearly grouped by 762-position on an UML tree (Figure 3A). While some organization of RNR classes within PolA groups emerged (e.g., Class I Cyano SP RNR and Tyr762 PolA), RNR classes were largely scattered across the tree and only showed distinct associations with undefined PolA sub-clades within the PolA 762 types. RNR amino acid sequences annotated by RNR class and the neighboring PolA 762 type showed four distinct clades according to RNR class on an UML tree (Figure 3B). Again, some organization of PolA 762 types within RNR classes (e.g., Class I Other RNR sequences distinctly associate with Phe762 and Tyr762 PolA genes) was observed, however, the more common observation was that PolA 762 types were scattered throughout RNR classes.

**FIGURE 4:**
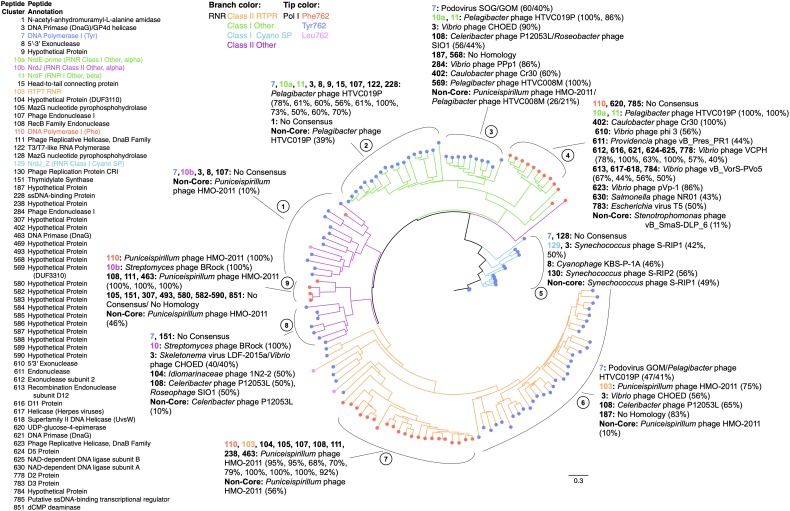
Concatenated gene analysis of PolA and RNR sequences provide a more parsimonious phylogeny. Unrooted maximum likelihood tree with 10 bootstrap replicates of PolA (*E. coli polA* gene A784-Q926) and RNR (*E. coli nrdA* gene N437-S625) concatenated peptide sequences. Branches are colored by RNR type and node tips are colored by PolA type (using the same color codes as for Figure 3). Nine distinct clades (brackets and circled numbers outside the tree) are labeled and occurred within four larger groups of clades defined by branch colors according to RNR Class (using the same color scheme as in Figure 3). Colored circles at the branch tips represent the PolA 762 type (using the same color scheme as in Figure 3). All peptides encoded on the 140 RNR-PolA contigs were clustered. A list of peptide cluster numbers and annotations for 60 frequently occurring peptide clusters is shown to the left of the phylogram. Adjacent to each clade label are peptide cluster numbers (in bold) that occurred in at least half of the contigs within a clade. These were considered “core” peptide clusters within the clade. Next to each bold peptide cluster number is the italicized name of a phage demonstrating significant BLAST homology (*E*-value ≤10^-5^) to the core peptide. Numbers in parentheses indicate the percentage of times a core peptide showed significant homology to the known phage gene. Overall, the Phe762 containing clades had a greater diversity of associated core peptide clusters and encoded distinct helicases as compared with the Tyr762 containing clades.

Concatenation of RNR and PolA genes enabled greater phylogenetic resolution of 140 SERC virome contigs encoding both PolA and RNR peptides (Figure 4). The concatenated tree revealed nine delineated clades based on the combined RNR class and PolA 762-type. Each of the four RNR classes occurring on the SERC contigs were separated on the tree with clades 1, 8, and 9 having a Class II Other RNR; clades 2, 3, and 4 having a Class I Other RNR; clade 5 having a Class I Cyano SP RNR; and clades 6 and 7 having a Class II RTPR. The combination of PolA 762-type with RNR class, clearly defined nearly all of the clades. Clades 2 and 3 were a combination of a Class I Other RNR with a Tyr 762 PolA; whereas, Clade 4 had a Phe762 PolA. Clade 5 had a Class I Cyano SP RNR with a Tyr762 PolA. Clade 6 had a Class II RTPR RNR with a Tyr762 PolA; whereas, Clade 7 had a Phe762 PolA. Clade 8 had a Class II Other RNR with a Tyr762 PolA; whereas Clade 9 had a Phe762 PolA. Lastly, Clade 1 was the only clade to indicate a mixture of PolA 762-types with a Class II Other RNR. Eleven contigs within Clade 1 had a Tyr762 PolA, one had a Phe762 PolA, and one had a Leu762 PolA.

All of the genes contained in the 140 contigs containing both PolA and RNR were clustered using BLASTp (*E*-value ≤1e-5) producing 2,179 gene clusters. Gene clusters occurring on at least half of the contigs within a clade were considered core gene clusters of a given clade. Altogether 60 gene clusters were identified as core genes. No genes were universally identified across the RNR-PolA clades (Figure 4). Clades 3, 6, and 8 contained contigs carrying Tyr762 PolA (Figure 2) and shared core genes DNA Primase (DnaG)/Gp4d Helicase (cluster 3), and RecB Family Endonuclease (cluster 108). These genes also displayed homology with similar reference sequences across clades; yet, these related clades were distinguished by different RNR genes (e.g., Class I Other RNR in clade 3 and Class II RTPR RNR in clade 6) (Figure 4). Similarly, clades 7 and 9 contained contigs carrying Phe762 PolA with MazG nucleotide pyrophosphohydrolase (cluster 105), RecB Family Endonuclease cluster 108), DnaB helicase (cluster 111), and DnaG DNA Primase (cluster 463) core genes, which were most similar to *Puniceispirillum* phage HMO-2011. Once again, these related populations were distinguished by different RNR genes (class II RTPR for clade 7 vs Class II Other for clade 9) (Figure 4). Therefore, the combination of PolA and RNR more accurately differentiated podoviral diversity in the most abundant phage groups (Tyr762 and Phe762 Group I, see above) than either marker gene alone.

## Discussion

Because polymerases are critical to viral replication, these genes may have a disproportionately important role in shaping the evolutionary history and fitness of the viruses that carry them (Doublie et al., 1998; Choi, 2012; Gimenes et al., 2012; Wommack et al., 2015). This study identified over 3,000 PolA sequences from aquatic viral metagenomes distributed around the globe, highlighting the ubiquity of this gene product within viral populations (Figure 1). However, in some cases, the biochemistry of PolA paralleled geographic and seasonal disparities among aquatic sites. Specifically, there was a high diversity of putative temperate phage, as defined by the Leu762 PolA, which were unique to the SERC dataset (Figure 1). This may reflect the intimate nature of phage-host interactions that give rise to specific and diverse phage populations. Moreover, previous studies have reported that lysogeny tends to increase and lytic populations tend to decrease in the winter months (Williamson et al., 2002). Since the SERC sample was obtained in December, the sampling season may be a contributor to the rise in diverse temperate phage. While this information is pertinent in regard to characterizing the difference in phage populations among environments, the single gene approach may provide only a snapshot of the information that can be drawn from metagenomic assemblies.

### Read Length Matters

By using a combination of long and short read technologies along with deep sequencing we were able to assemble full-length *polA* genes and produce longer contigs from a single virome (Supplementary Table S1). The hybrid assembly of long and short sequence reads produced a collection of full- and partial-length PolA peptides representing 69% of known viral PolA diversity and expanding it by ca. 80% (Figure 1). The longer contigs resulting from the hybrid assembly revealed the gene neighbors flanking PolA. Not surprisingly, genes commonly occurring with PolA were those related to DNA replication, such as helicases and RNRs (Figure 2). This is in agreement with well-known model bacteriophages of *E. coli* (e.g., λ, T4, T7) and *Bacillus subtilis* (e.g., SPP1, ϕ29) (Alonso et al., 1997; Doublie et al., 1998; Miller et al., 2003). These single genome experiments found that genes encoding replication functions were positioned close to one another, resulting in “replication modules” (Weigel and Seitz, 2006). A more recent study of over 1,000 dsDNA viral genomes found that DnaB-like helicases, as well as, Polymerase A and DnaG-like primases dominated the representative bacteriophage genomes (Kazlauskas et al., 2016). In this genomic analysis all of the viral genomes with a DNA polymerase (family A, B, or C) also included a replicative helicase indicating both the close biochemical interaction of DNA polymerase and helicase. It is likely this intimate interaction that prevents the phage polymerase from utilizing any host helicases for replication of the phage genome. As shown in our analysis DNA PolA 762 mutant types may be associated with specific helicases within the larger replication module.

### Helicase Structure Complements Phage Lifestyle

Helicases are motor proteins driven by the hydrolysis of 5′-nucleoside triphosphates (NTP) to unwind nucleic acids for processes vital to phage production, such as replication, recombination, and repair. Therefore, the structure and biochemistry of a helicase within a phage genome may be connected to PolA and RNR biochemistry and more broadly predictive of phage lifestyle. Ring shaped helicases (e.g., Gp4, DnaB) were largely associated with PolA genes in Phe762 Group I and Tyr762 groups, whereas Leu762 PolA groups contained non-ring oligomeric helicases (e.g., UvrD, PcrA). Ring shaped helicases generally function in a 5′–3′ polarity by enclosing the nucleic acid around a central channel and catalyzing strand displacement (Patel and Donmez, 2006). This enables them to be more processive than helicases like UvrD and PcrA. For example, the ringed helicase of coliphage T7 (Gp4) has been reported to translocate along single-stranded DNA (ssDNA) an average of about 75 Kbp before dissociating (Kim et al., 2002). In comparison, the non-ring oligomeric helicase, UvrD, unwinds dsDNA in about 10 discrete steps (∼4–5 bp) before dissociating (Ali and Lohman, 1997). As a result, ring shaped helicases would be advantageous to a lytic lifestyle where high processivity would lead to rapid production of phage progeny. A prior metanalysis of phenomic connections to PolA 762 mutant types found that Tyr762 and Phe762 Group I PolA peptides were only found within phages demonstrating a lytic lifecycle (Schmidt et al., 2014). Thus, it makes sense that highly processive ringed helicases such as Gp4 and DnaB were both found in association with these polymerase groups found in lytic phages (Figures, 2, 4).

Conversely, non-ring oligomeric helicases, seen in the Leu762 PolA groups, may be more beneficial to a temperate lifestyle due to the fact that RecB and UvrD have 3′–5′ helicase polarity and function in DNA recombination and repair (Oeda et al., 1982; Boehmer and Emmerson, 1991; Yu et al., 1998; Tuteja and Tuteja, 2004). This increase in helicase fidelity may compliment the slower, more faithful, PolA observed with the Leu762 mutation (Suzuki et al., 2000), a mutation that has been observed in cultivated temperate phage (Schmidt et al., 2014).

### Ribonucleotide Reductase

Similar to PolA, RNR has been estimated to occur in at least 17% of all known dsDNA phage genomes (Wommack et al., 2015). RNRs are particularly prevalent among virulent *Myoviridae* (T4-like), *Siphoviridae* (*Mycobacteriophage smegmatis* infecting), and *Podoviridae* (cyanophage and N4-like) (Wommack et al., 2015). RNRs are vital to DNA synthesis, reducing ribonucleotides to deoxyribonucleotides and, thus, controlling the overall rate of DNA replication through the supply of available substrates for DNA synthesis (Lundin et al., 2010). RNRs are abundant in aquatic viral metagenomic libraries and have been previously used to characterize the diversity of viral populations (Dwivedi et al., 2013; Sakowski et al., 2014). In this study RNRs were commonly observed within the genetic neighborhood of Phe762 Group I and Tyr762 PolA genes but were rarely associated with PolA genes in the Leu762 groups (Figures 2, 4). RNRs have been previously reported to be prevalent in lytic marine phage (Sakowski et al., 2014). A literature survey of 204 RNR-containing phages (Wommack et al., 2015) found that 193 were virulent, two were pseudolysogenic, and nine were unpublished other than their whole genome sequence (Supplementary File S3). Moreover, the common observation of RNRs and ringed helicases associated with Phe762 and Tyr762 PolAs and the rare observation of RNRs associated with Leu762 PolAs lends further support to the hypothesis that Tyr762 and Phe762 PolA groups belong to virulent phages within the virioplankton. Among the 140 SERC contigs containing both PolA and RNR, significant homology to genome sequences within known lytic podoviruses infecting hosts within abundant marine bacterial lineages were common. Among these phages *Pelagibacter* phage HTVC019P infecting SAR11 hosts (Zhao et al., 2013) and *Puniceispirillum* phage HMO-2011 infecting SAR116 hosts (Kang et al., 2013) commonly showed significant homology to RNR-PolA containing SERC contigs (Figure 4).

### Other Replisome Genes

There were several other genes identified among the core replicative modules of RNR and PolA containing contigs (Figure 4). For instance, DNA Primase (DnaG) was identified in all the RNR and PolA containing clades. Primases synthesize short ssRNA or ssDNA segments, which are then used as primers by the DNA Polymerase during replication. DnaG primases, in particular, are closely associated or fused with Gp4 and DnaB helicases (Ilyina et al., 1992), with their interactions playing a fundamental role in the initiation and rate of DNA replication. In *Bacillus stearothermophilus*, the interaction between DnaG and DnaB increases the nucleoside triphosphatase and helicases activities (Bird et al., 2000) of the cell. DnaG, added to a reaction mixture of purified bacteriophage SPP1 hexameric helicase (G40P), increased helicase activity by threefold (Ayora et al., 1998). DnaG primase (cluster 3, Figure 4) occurred as a core protein cluster in five of the nine clades identified on the concatenated RNR-PolA tree. Thus, the occurrence of DnaG along with RNR and a Tyr762 PolA on a single contig may be predictive of a fast-replicating lytic phage population.

Additionally, certain genes were unique to particular clades on the concatenated RNR-PolA tree (Figure 4). While these genes may not be members of the core ancestral replication modules, they may speak to the environmental specificity of phage populations. For example, MazG nucleotide pyrophosphohydrolase was identified on clade 5 contigs (Class I Cyano SP RNR; Tyr762 PolA), clade 7 contigs (Class II RTPR; Phe762 PolA), and was a core gene on clade 9 contigs (Class II Other RNR; Phe762 PolA). In *E. coli* MazG was found to hydrolyze guanosine 3′, 5′ bispyrophosphate (ppGpp), an inhibitor of RNA synthesis during times of amino acid starvation (Gross et al., 2006). Within cyanophages MazG is also believed to be used during amino acid starvation by reducing the amount of ppGpp, thereby sustaining phage transcription/replication (Bryan et al., 2008). This auxiliary metabolic gene was thought to occur within several marine cyanophage by means of horizontal gene transfer (Bryan et al., 2008). For RNR-PolA clades where MazG is pervasive within the replication module, nutrient starvation may have selected for phage populations that could optimize the physiology of its hosts by reducing the effects of ppGpp and stabilizing the rate of RNA synthesis.

### Compiling a Field Guide for Aquatic Viral Metagenomics

Environmental viruses likely possess the majority of genetic diversity on the planet (Paez-Espino et al., 2016). This makes it difficult to study natural viral assemblages, as many predicted genes from viromes and viral genomes have not been seen more than once. Collectively these genes have been termed the “viral dark matter” (Roux et al., 2015). Many individual factors such as recombination, mutation, and fast replication rates contribute to the high genetic diversity of viruses; however, the overarching reason for this diversity is the ubiquity and extraordinary abundance of viruses within ecosystems. Because of their ubiquity and abundance, even the rarest of genetic events such as non-homologous recombination occur millions of times a day within the viral realm (Pedulla et al., 2003).

**FIGURE 5:**
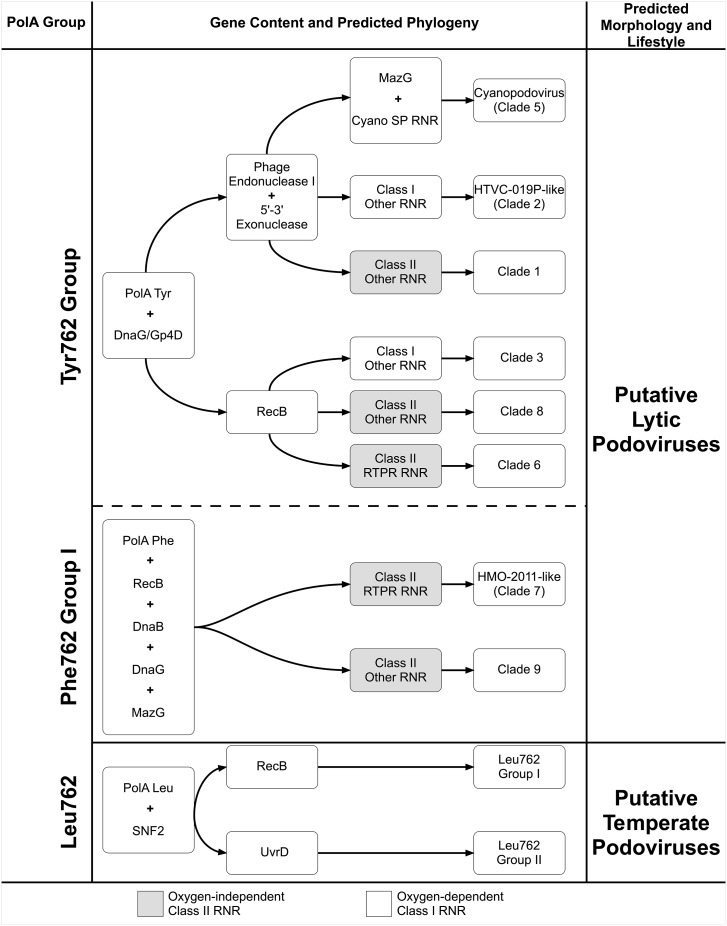
Virome “field guide” for phage based on DNA PolA and other replisome genes. The dichotomous key summarizes the genome to phenome connections that have been described throughout the manuscript and predicts phage phylogeny based on replisome gene content. Phenome characterization and phylogeny prediction begins with DNA PolA type (Tyr762, Phe762 Group I and Leu762 groups) and associated “core” genes (i.e., genes observed in ≥50% of contigs; far left). Phage populations are then differentiated by nuclease/helicase identity and RNR type (if applicable). Finally, phage populations and lifestyle are predicted (far right). Clade predictions refer to those clades identified in the PolA-RNR concatenated tree in Figure 4. Leu762 groups correspond to the clades described in Figure 1.

Patterns in viral gene content routinely emerge from virome studies, despite the staggering degree of viral diversity, due to selective pressures (Brum et al., 2016). An ideal analysis of a viral community from virome data would indicate the catalog of genes and gene functions, measures of community structure and diversity, and include hypothetical predictions of the biology and ecology (i.e., the phenome) of unknown viral populations within an ecosystem. Of these analyses, predictions of the biology and ecology of viral populations have been the most elusive. Here, we propose the creation of “field guides” as a way to collect the knowledge-driven observations of genome to phenome connections that have been described in this manuscript (Figure 5). The guide presented here is rooted in the arrangement of nucleotide replication and metabolism genes and can be used to enhance the predictive capacity of virome data and reveal the role of viral processes in ecosystems. Such a guide could be particularly useful for inferring viral population biology and ecology from incomplete viral metagenome assemblies since only a few genes need be identified. Future field guides could target different genes and viral populations, shedding light on those genes most likely to drive phage diversity, biology, and ecology, as well as provide a comprehensive tool for developing a more holistic picture of viruses from fragmentary shotgun data.

## Summary

An expansive survey of 87 virome libraries spanning aquatic, organismal, and terrestrial ecosystems demonstrated that DNA polymerase A occurs within a diverse cross-section of viruses. Surprisingly, a single, deeply sequenced, virome library from the Chesapeake Bay encompassed a majority of the viral PolA diversity seen across the other 86 virome libraries and contained unique PolA genes not seen elsewhere. Long contigs resulting from the assembly of long and short reads from the Chesapeake Bay virome enabled extensive gene neighbor analysis of other nucleotide metabolism genes associated with PolA. The biochemical characteristics of helicases, ribonucleotide reductase, and primases associated with PolA revealed deeper clues as to the genome to phenome linkages within unknown virioplankton populations, an aspect of metagenomic surveys that is often overlooked. Finally, the gene content of viral replication modules was developed into a “field guide” for predicting the phenomic characteristics of unknown viruses based on metagenomic observations.

## Author Contributions

JC, ES, DN, SP, and KW designed the research. JC, DN, and ES performed the research. DN and JC wrote the paper. DN, JC, ES, BF, SP, and KW revised the paper.

## Conflict of Interest Statement

The authors declare that the research was conducted in the absence of any commercial or financial relationships that could be construed as a potential conflict of interest.

## Footnotes

1 http://metagenomesonline.org

2 github.com/dnasko/blasters
