# Supplementary material for: Family A DNA Polymerase Phylogeny Uncovers Diversity and Replication Gene Organization in the Virioplankton
Source: Front Microbiol. 2018 Dec 14;9:3053. doi: 10.3389/fmicb.2018.03053 (PMC6302109; doi:10.3389/fmicb.2018.03053)
Supplement: Supplementary file 4 [file Data_Sheet_1.docx]

**SUPPLEMENT**

**Supplementary File S1: Predicted PolA peptides from Metagenomes Supplementary File S2: Gene neighbor association by 762 group.**

**Table S1:** **Polymerase I peptides and replication components predicted from various sequencing technologies and assembly strategies.**

| Technology | Pol I ORFs | 75% Clusters | Replication Gene Neighbors | % With Replication Genes | Mean Contigs Length (bp) |
| --- | --- | --- | --- | --- | --- |
| **Pol I’s ≥ 200 AA** |  |  |  |  |  |
| PacBio | 138 | N/A | 99 | 72% | 8571 |
| Illumina | 1074 | N/A | 444 | 41% | 6537 |
| Hybrid | 2095 | N/A | 824 | 39% | 7675 |
| **Full-Length PolA’s** |  |  |  |  |  |
| PacBio | 53 | 12 | 37 | 70% | 11952 |
| Illumina | 435 | 79 | 275 | 63% | 10060 |
| Hybrid | 830 | 81 | 571 | 69% | 12671 |

**Table S2: Breakdown of PolA and RNR classifications on virioplankton contigs containing both genes.**

| Pol I Type | RNR Type | Count |
| --- | --- | --- |
| Phe | Class II Other | 6 |
| Phe | Class II RTPR | 22 |
| Phe | Class I Other | 11 |
| Leu | Class II Other | 1 |
| Leu | Class II RTPR | 2 |
| Tyr | Class I Cyano SP | 14 |
| Tyr | Class II Other | 18 |
| Tyr | Class II RTPR | 38 |
| Tyr | Class I Other | 28 |
